# Loading-related regulation of gene expression in bone in the contexts of estrogen deficiency, lack of estrogen receptor α and disuse

**DOI:** 10.1016/j.bone.2009.10.021

**Published:** 2010-03

**Authors:** Gul Zaman, Leanne K. Saxon, Andrew Sunters, Helen Hilton, Peter Underhill, Debbie Williams, Joanna S. Price, Lance E. Lanyon

**Affiliations:** Department of Veterinary Basic Sciences, The Royal Veterinary College, University of London, Royal College Street, London NW1 0TU, UK

**Keywords:** Gene expression, Mechanical loading, Bone, Estrogen, ERα

## Abstract

Loading-related changes in gene expression in resident cells in the tibia of female mice in the contexts of normality (WT), estrogen deficiency (WT-OVX), absence of estrogen receptor α (ERα^−/−^) and disuse due to sciatic neurectomy (WT-SN) were established by microarray. Total RNA was extracted from loaded and contra-lateral non-loaded tibiae at selected time points after a single, short period of dynamic loading sufficient to engender an osteogenic response. There were marked changes in the expression of many genes according to context as well as in response to loading within those contexts. In WT mice at 3, 8, 12 and 24 h after loading the expression of 642, 341, 171 and 24 genes, respectively, were differentially regulated compared with contra-lateral bones which were not loaded. Only a few of the genes differentially regulated by loading in the tibiae of WT mice have recognized roles in bone metabolism or have been linked previously to osteogenesis (*Opn*, *Sost*, *Esr1*, *Tgfb1*, *Lrp1*, *Ostn*, *Timp*, *Mmp*, *Ctgf*, *Postn* and *Irs1*, *BMP* and *DLX5*). The canonical pathways showing the greatest loading-related regulation were those involving pyruvate metabolism, mitochondrial dysfunction, calcium-induced apoptosis, glycolysis/gluconeogenesis, aryl hydrocarbon receptor and oxidative phosphorylation. In the tibiae from WT-OVX, ERα^−/−^ and WT-SN mice, 440, 439 and 987 genes respectively were differentially regulated by context alone compared to WT. The early response to loading in tibiae of WT-OVX mice involved differential regulation compared to their contra-lateral non-loaded pair of fewer genes than in WT, more down-regulation than up-regulation and a later response. This was shared by WT-SN. In tibiae of ERα^−/−^ mice, the number of genes differentially regulated by loading was markedly reduced at all time points.

These data indicate that in resident bone cells, both basal and loading-related gene expression is substantially modified by context. Many of the genes differentially regulated by the earliest loading-related response were primarily involved in energy metabolism and were not specific to bone.

## Introduction

The observation that bone mass and architecture are profoundly influenced by their loading experience to produce structures apparently better suited to resist such loading without damage is one of the oldest in modern biology [1,2]. Paradoxically the clinical significance of the relationship in humans increases as, in affluent Western societies at least, habitual exposure of the skeleton to substantial load bearing declines. One correlate of this decline, which may indeed be partially a consequence, is the increasing incidence of fractures resulting from skeletal fragility [3,4].

Skeletal architecture is the product over time of coordinated activity of populations of cells which form and resorb bone (osteoblasts and osteoclasts respectively). The osteoblast population (and their derivates the osteocytes) reside in close proximity to the matrix which is responsible for carrying functional loads. The stimulus used by this population of cells to control structurally appropriate modeling and remodeling of their matrix is assumed to be derived from the strains that functional load-bearing engenders [5,6]. Despite the importance of this relationship the mechanisms by which resident bone cells transduce mechanical information into biochemical responses and then process it into signals controlling the modeling and remodeling behaviour of osteocytes, osteoblasts and osteoclasts, is obscure.

Mechanical loading is not the only stimulus to which bone cells respond since they are exposed to all the other nutritional, endocrine, paracrine and autocrine stimuli inherent to their situation *in vivo*. They are particularly responsive to estrogen, to an extent that seems unnecessary for the discharge of this hormone's primarily reproductive responsibilities. Associated with this responsiveness, there is a rapid loss of bone in women at the time of estrogen withdrawal at the menopause. The level of bone loss in women at the menopause and the subsequent slower, progressive age-related loss that occurs in both men and women are substantially related to their levels of bio-available estrogen [7–10]. Despite recognition of this association, the mechanisms by which reduction in circulating estrogen levels account for this bone loss have never been satisfactorily explained. In contrast the estrogen receptor (ER), specifically ERα, has been shown to be involved in bone cells' early responses to strain both *in vitro* [11–13] and *in vivo* [14]. In humans, genetic variation in ERα is associated with different responses to load-bearing exercise [15,16]. ERα is also one of the genes in which variation is associated with the severity of osteoporosis [17]. In mice when ERα is absent the adaptive response to artificial loading *in vivo* is attenuated [14]. The levels of ERα in osteoblasts and osteocytes are regulated by estrogen but not by strain [18] so the down-regulation of ERα associated with low levels of estrogen may reduce the effectiveness of bone cells' ERα-mediated responses to strain [19,20]. This would allow reduction of bone mass with its associated increases in fragility, functional strains and fracture incidence.

Although these studies provide a possible explanation for how the reduced effectiveness of bone cells' osteogenic response to bone loading could be associated with estrogen-related decline in ERα, the underlying cellular and molecular mechanisms remain to be elucidated. Microarray technology provides a means to examine simultaneously the gene expression of the entire transcriptome in a single sample of bone. In this article, we describe experiments designed to establish the pattern of gene expression in resident cells of the tibia of normal female wild-type (WT) mice following a single period of *in vivo* loading with demonstrated capacity to stimulate osteogenesis. The pattern of gene expression following such a single period of “osteogenic” loading should be the gene regulation “signature” of the stimulus for the osteogenic response. To establish that “signature”, we compared the pattern of gene expression in bones that had recently experienced a single, short period of artificial loading with that from their contra-lateral controls that were only exposed to habitual, natural loading.

We then established the differences from this “signature” arising from three relevant contexts: estrogen deficiency, lack of ERα and disuse. Estrogen deficiency and disuse were achieved in WT C57BL/6 mice by ovariectomy (WT-OVX) and unilateral sciatic neurectomy (WT-SN), respectively. Absence of ERα was achieved by using an ERα knockout mouse on a C57BL/6 genetic background [21].

## Materials and methods

### Experimental animals

Female C57BL/6 (WT) mice were purchased from Charles River Laboratories (Margate, Kent, UK). Ovariectomy and sciatic neurectomy were performed on WT mice at 11 weeks of age. ERα null mutant mice (ERα^−/−^) [21] were a gift from Ken Korach. The model was created by targeting ERα gene in ET12TG2a ES cells and injecting the targeted cells into C57BL/6 blastocytes. Resultant chimeras were backcrossed to C57BL/6 for 10 generations. The poor breeding performance of the ERα^−/−^ mice meant that we were unable to use extensive backcrossing to ensure that the two breeding populations were maintained as genetically similar as possible. Thus, it must be remembered that differences in gene regulation between these two populations could be contributed to by genetic drift as well as the presence or absence of ERα. All experiments were approved by the Royal Veterinary College's ethics committee and by the UK Home Office.

### Mechanical loading of the mouse tibia *in vivo*

To firstly establish the osteogenic response of a single loading session, the right tibiae of six female WT mice were exposed to a single period of loading in sine wave form producing a peak strain of − 1300 microstrain at a frequency of 2 Hz for 30 s (60 cycles). The contra-lateral limb was not loaded and served as an internal control. The apparatus and protocol for dynamically loading the mouse tibia were essentially the same as reported previously [22, 23].

Bone formation was assessed from the distribution of two calcein labels (7 mg/kg; Sigma-Aldrich Company Ltd., Gillingham, UK) injected intraperitoneally on the day of loading and 48 h later. Mice were euthanized on the 4th day after loading. The tibiae from these animals were processed as described earlier [24]. The calcein labels were read using a laser scanning confocal microscope (Carl Zeiss MicroImaging, GmbH, Gena, Germany).

In the non-loaded control tibiae of the six WT mice, the cross-sectional area of bone deposited in the 48 h between the two labels at 37% along the bone shafts from the proximal end of the tibia was 588 ± 124 μm^2^. In the contra-lateral tibiae of these mice, which were exposed to a single period of dynamic axial loading, the area of new bone at the same site was 873 ± 127 μm^2^; a statistically significant increase of 48 percent (*p* < 0.05).

For the microarray experiment, the right tibiae of 15-week-old female WT (*n* = 32), ovariectomized (WT-OVX, *n* =  32), neurectomized (WT-SN, *n* =  40) and ERα^−/−^ mice (*n* =  24) were subjected to a single period of dynamic axial load using a hydraulic actuator under feedback control (Dartec HC10, Zwick Testing Machines Ltd., Leominster, UK). The load was applied during this single period of loading in a sine waveform, at a frequency of 2 Hz for 30 s (60 cycles). The peak load magnitude for each group was adjusted to produce a peak compressive strain of − 1300 microstrain measured by longitudinally aligned strain gauges attached to the medial mid-shaft of separate “calibration” mice. For WT and ERα^−/−^ mice, the peak load necessary to achieve a peak strain of − 1300 microstrain was 12 N and for the WT-OVX and WT-SN mice 8 N.

In the WT-SN group, a separate group of mice were sciatic neurectomized and the tibiae in their neurectomized hindlimbs used as non-loaded controls. In the other groups (WT, WT-OVX and ERα^−/−^), the contra-lateral limb was not loaded and this served as an internal control. In every case, mice whose tibiae were to be loaded were anesthetized with halothane for approximately 3 min during which loading took place. They were allowed to recover immediately after loading. Eight animals were sacrificed at each time point of 3, 8, 12 and 24 h after loading except for mice in the ERα^−/−^ group where shortage of animals meant that sampling at only 3, 8 and 24 h was possible.

A recent report by Sample et al. [25] suggests that vigorous loading of the rat ulna on one side produces regional and systemic changes in remodeling of adjacent bones and those in the contra-lateral limb. To confirm that this was not the case in our experiments, we have compared the remodeling in left and right non-loaded bones of mice to that in bones contra-lateral to those which have been loaded. We detect no difference in the remodeling in these contra-lateral bones from that in either left or right bones in individuals that have experienced no loading [26]. From this we are confident that in our experiments the (re)modeling response to loading and the changes in gene expression that precede and control it are confined to the bone actually loaded. Thus, the use of the non-loaded contra-lateral bone as a control is valid.

### Total RNA isolation from mouse bones

The right and left tibiae were carefully dissected and all their surrounding musculature removed leaving the periosteum intact. The cartilaginous ends of the bones were removed and the remaining tibial shaft spun at 5000 rpm for 2 min (Eppendorf centrifuge) to remove the marrow. The tibial shafts were then snap-frozen in liquid nitrogen, pulverized under liquid nitrogen using a mortar and pestle and lysed in Qiazol lysis reagent (Qiagen Ltd., Crawley, UK). Total RNA from these lysed samples was purified and DNase treated using RNeasy Mini Kit (Qiagen) according to the manufacturer's protocol. The quantity and the integrity of the purified RNA was assessed using the Agilent RNA Bioanalyzer (Agilent Technologies UK Limited, Stockport, UK).

### cDNA preparation

Double-stranded cDNA was synthesised from the tibial shaft-derived total RNA (1 μg) using a SMART protocol (Clontech-Takara Bio Europe, 78100 Saint-Germain-en-Laye, France) [27]. Two microliters of this first strand reaction was used in the amplification step which was performed using 17 rounds of cycling. The double-stranded cDNA was checked on a 1.2% agarose gel before the samples were purified using QIAquick clean up columns (Qiagen). The amount of cDNA amplified was then checked using the NanoDrop ND1000 spectrophotometer (Labtech International, Ringmer, East Sussex, UK). For the reference cDNA, a pool containing 1 μg of each of the individual RNA samples was used. This RNA pool was then used to prepare cDNA using the SMART protocol (as above). The cDNA from the second strand reactions were then pooled, thus ensuring that the same reference cDNA was used in every hybridization. A common reference design scheme enabled any sample to be compared with any of the other samples.

### cDNA labelling and microarray hybridization and scanning

Using the Bioprime labelling kits (Invitrogen Ltd., Paisley, UK), 750 ng of purified cDNA was labelled by incorporating 2 μl of Cye dye (GE Healthcare, Little Chalfont, Bucks, UK). After incubating for 3 h at 37 °C, the labels were purified using ProbeQuant G50 micro columns (GE Healthcare). Incorporation rates were determined using a NanoDrop ND1000 spectrometer before specific labels were pooled and dried down to completion. The labels were re-suspended in 40 μl of hybridization buffer (40% deionised formamide; 5× Denhart's; 5× SSC; 1 mM Na pyrophosphate; 50 mM Tris pH 7.4; 0.1% SDS) and hybridized onto an RNG-MRC mouse set 25K microarray printed on GE Codelink slide (http://www.har.mrc.ac.uk/services/MPC/microarray/), overnight at 48 °C in a water bath using the Corning hybridization chambers. Three replicate hybridizations were performed for each sample. After hybridization, the arrays were washed initially in 2× SSC until the coverslip had come off, then for 5 min with vigorous shaking in 0.1× SSC, 0.1% SDS and finally in 0.1× SSC for 2 min with further vigorous shaking. The arrays were then spun dry and scanned using a ProScanArray HT (PerkinElmer, Maidenhead, UK) at 7 different photomultiplicator (PMT) gain settings from 40 to 70. The images were then processed using ImaGene 6.0.1 (Bio Discovery, El Segundo, CA, USA).

### Analysis of microarray

Global gene expression was examined in 27 different RNA pools from loaded and corresponding non-loaded mouse tibiae as well as a reference pool (containing 1 μg of each of the individual 27 RNA samples) using an oligonucleotide array consisting of 25,000 target genes. The scanned images were processed using ImaGene 6.0.1 (BioDiscovery, PerkinElmer), where all images from a single array were overlaid and gridded and the feature data extracted. These data were then further processed using Mavi 2.6.0 (MWG Biotech AG, Ebersberg, Germany), which increases the dynamic range while avoiding saturation problems. The software performs a linear regression analysis on each gene across the increasing PMT gains to detect any saturation or degradation of signal and returns the value at a set PMT for all genes, giving a single data set from the 7 multi-gain sets for each array. The data were then loaded into R Project for Statistical Computing (http://www.r-project.org) for further analysis. A two-dimensional loess normalization, from the YASMA5 (Yet Another Statistical Microarray Analysis) library, was performed on each array to correct for any spatial variation within the slide. The LIMMA [28] library (Linear Model for Microarray Analysis) from the BioConductor (http://www.bioconductor.org) software project was used further to normalize the data and to select differentially expressed genes. In brief, a linear model is fitted to the data for each gene to fully model the systematic part of the data and provide estimates for each coefficient (samples in this case). These coefficients can then be compared and differentially expressed genes selected using an empirical Bayes moderated *t*-statistic. Data were loaded into LIMMA and control spots were zero-weighted, so the slides were normalized within slide using loess and then normalized across the slide using a quantile normalization only on the reference sample for each array [29]. Each array was then given a weight based on how well it fits the linear model. This was then used during the model fitting stage [30]. A contrast matrix comparing every sample to every other sample was fitted to allow all possible comparisons to be made. Differential genes were selected for the comparisons of interest based on their moderated *t*-statistic [31] after using a false discovery rate control of 5% [32]. Fitted values for each sample were then loaded into GeneSpring GX (Agilent Technologies, Stockport, UK) to allow for easy comparison of lists of differential genes. These lists were further filtered using a Grubbs Test to remove genes with high variance in the control replicates.

### Bioinformatic analysis of differentially expressed genes

We used Ingenuity Pathways Analysis (IPA, http://www.ingenuity.com) to arrange the gene expression data according to complexes, canonical signaling pathways and networks. To do this, we uploaded the data of genes differentially expressed by context into IPA and mapped these data to their corresponding genes in the Ingenuity knowledge base. The canonical pathway analysis identified the pathways from the IPA library that were most significant to each data set. The *p*-value, evaluated using the right-tailed Fisher's exact test, of each pathway was calculated by comparing the number of user specified genes of interest (i.e., differentially regulated genes) that participated in a given pathway relative to the total number of occurrences of these genes in all pathway annotations stored in the Ingenuity knowledge base. The functional analysis identified functions that were most significant to the data set. Heat map visualization of clusters formed was generated by using CLADIST (Integrated Cluster and Interaction and Transcriptional Networks Analysis Tool) developed by LICR-Bioinformatics Group. CLADIST is available at *http://pstiing.licr.org/*). Hierarchical clustering analysis was done in Euclidean distance by average linkage method.

### Quantitative real-time RT-PCR

For validation of microarray data, quantitative real-time RT-PCR (qRT-PCR) was used to measure changes in expression of selected genes. One microgram of the total RNA from loaded and control tibiae was reverse transcribed with Superscript II Reverse Transcriptase (Invitrogen). Primer sequences, the positions in the coding region, the expected qRT-PCR products and the accession number for the genes of interest and the housekeeping gene are summarized in Table 1. QuantiTect SYBR Green PCR kit (Qiagen) and Opticon 2 Lightcycler (MJ Research, Waltham, MA, USA) were used to perform qRT-PCR. A standard curve was constructed for each gene of interest and the housekeeping gene and these standards were included in each run. Standards were run in duplicates and samples in triplicates. Samples of unknown concentration were quantified relative to their standard curve. Gene expression levels were normalized to the housekeeping gene β-actin (*Actb*).

### Statistical analysis

In addition to the statistical analysis described in the bioinformatics methods section, the significance of differences in inter-label areas between non-loaded and loaded groups was determined by using paired Student's *t*-test. Results were considered significant at *p* <  0.05.

## Results

### The effect of context on gene expression

#### The number of genes differentially regulated

**Fig. 1:**
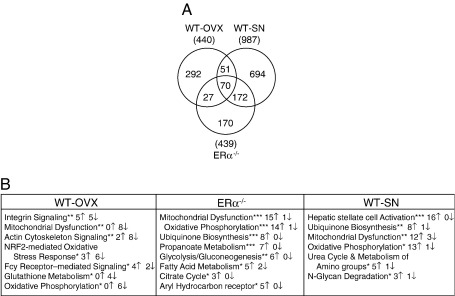
Effect of context on gene expression in non-loaded mouse tibia at the  3-h time point. (A) A Venn diagram of the number of differentially expressed genes in tibiae from WT-OVX, ERα^−/−^ and WT-SN mice backgrounds in comparison to WT mice tibiae. Total RNA was extracted from the non-loaded mouse tibiae at the  3-h time point. The numbers in brackets, beneath the treatment group name, represent the total number of genes differentially regulated in that group in comparison to WT mice tibiae. (B) Canonical pathways most significant to each set of these differentially expressed genes in tibiae from WT-OVX, ERα^−/−^ and WT-SN mouse backgrounds as analyzed by Ingenuity Pathway Analysis software. The canonical pathways shown are ranked with the most significant pathway heading the list in each context. The significance is based on a Fisher's exact test as described in the methods (⁎*p* <  0.05, ⁎⁎*p* <  0.01 and ⁎⁎⁎*p* <  0.001). The number of genes up- and down-regulated in each canonical pathway within each context are also illustrated.

Comparison of the numbers of genes differentially expressed in non-loaded tibiae of the WT with those in the other groups, using GenBank accession number as the unique identifier, showed differential regulation in a large number of genes according to context. The number of genes and their overlap in relation to context is shown in Fig. 1A. In the tibiae from WT-SN mice, 987 (694 + 172 + 70 + 51) genes were differentially expressed compared to WT, in the tibiae from WT-OVX mice 440 (292 + 51 + 70 + 27) and in those from ERα^−/−^ mice 439 (170 + 172 + 70 + 27) (Fig. 1A). Complete lists showing the effect of context (WT-OVX, ERα^−/−^ and WT-SN) on differential gene expression are provided in the data (Table CL1) posted at the Royal Veterinary College website (http://www.rvc.ac.uk/files/zaman2009microarraystudy).

Of the 440 genes differentially regulated by OVX, the 1.6-fold increase in ERα (*Esr1*) mRNA levels is of particular interest (Table S1). It agrees with previous findings in trabecular bone where ovariectomy was shown to reduce levels of ERα protein but elevate ERα mRNA [33]. In our present study, OVX resulted in reduced expression levels of some genes previously associated with bone cells (integrin-alpha 1 (*Itga1*, − 2.1-fold), *Itga4* (− 2.0-fold), heme oxygenase-1 (*Hmox1*, − 1.8-fold) and transforming growth factor beta 1 (*Tgfb1,* − 1.9-fold)) and others (nobox oogenesis homeobox (*Nobox,* − 7.8-fold) and titin (*Ttn,* − 2.4-fold)). Expression of the genes for insulin receptor substrate-1 (*Irs1*), creb-binding protein (*Crebbp*) and bone morphogenetic protein-4 (*Bmp4*) was up-regulated in tibiae from WT-OVX mice (2.6-, 2.1- and 1.6-fold, respectively).

Of the 439 genes differentially regulated in the tibiae of ERα^−/−^ mice, a number of genes required for differentiation of adipocytes and the subsequent maintenance of their phenotype were up-regulated (CCAAT/enhancer-binding protein (*Cebpd*, 1.4-fold), acyl-coenzyme oxidase 1(*Acox1*, 1.9-fold) and fatty acid-binding protein 3 (*Fabp3*, 2.1-fold)) (Table S2) as were striatin (*Strn*, 10.1-fold), peroxisome proliferative activated receptor gamma coactivator 1 alpha (*Ppargc1a*, 2.1-fold), myocyte enhancing factor-2a isoform 1 (*Mef2a*, 1.8-fold), integrin-beta 6 (*Itgb6*, 2.1-fold), integrin-beta 4 (*Itgb4*, 1.6-fold), myogenic factor 6 (*Myf6*, 2.9-fold), connective tissue growth factor (*Ctgf*, 2.0-fold), *Ttn* (1.7-fold), osteocrin (*Ostn*, 1.7-fold), caveolin-1 (*Cav1*, 1.7-fold), *Cav2* (1.7-fold) and *Cav3* (1.5-fold). Among the genes which were down-regulated were low-density lipoprotein receptor-related protein 5 (*Lrp5*, − 1.8-fold), acid phosphatase 5, tartrate resistant (*Acp5*, − 1.7-fold) and *Tgfb1* (− 1.8-fold).

Sciatic neurectomy was associated with up-regulation of a number of genes associated with Wnt/β-catenin signaling (*Lrp1*, 1.8-fold), *Lrp4* (1.8-fold), Wnt-inducible signaling protein 1 (*Wisp1*, 1.7-fold), *Wisp2* (2.2-fold), secreted frizzled-related protein 1 (*Sfrp1*, 2.2-fold), *Sfrp2* (1.8-fold), *Sfrp4* (2.0-fold) and dickkopf-3 (*Dkk3,* 1.7-fold) (Table S3). Other genes of interest up-regulated in WT-SN mouse tibiae include insulin-like growth factor II receptor (*Igf2r*, 2.4-fold), *Ctgf* (1.7-fold), growth differentiation factor 10 (*Gdf10*, 1.5-fold), fibroblast growth factor 8 (*Fgf8*, 1.5-fold), ERα (*Esr1*, 1.8-fold), early growth response 1 (*Egr1*, 2.2-fold), *Myf6* (7.4-fold), periostin (*Postn*, 2.4-fold), ankyrin repeat domain 1 (*Ankrd1*, 7.6-fold), *Ostn* (1.7-fold), osteoglycin (*Ogn*, 2.5-fold), distal-less homeobox 5 (*Dlx5*, 1.8-fold), integrin-beta 4 (*Itgb4*, 1.8-fold), tenascin (*Tnn*, 2.1-fold) and Bcl-2/E1B 19-kDa interacting protein 3 (*Bnip3*, 2.1-fold). Five genes coding for matrix metalloproteinases (MMPs) were also up-regulated. A large number of genes involved in adhesion were markedly up-regulated in the tibiae of WT-SN animals including fibronectin 1 (*Fn1*, 4.0), *Postn* (2.4-fold), *Itgb4* (2.0-fold), *Tnn* (1.8-fold), fibulin 2 (*Fbln2*, 3.0-fold), laminin-alpha 4 (*Lama4*, 2.4-fold), lambin-beta 1, subunit 1 (*Lamb1-1*, 2.4-fold), glycoprotein 38 (*Gp38* (4.0-fold) and thrombospondin 2 (*Thbs2*, 3.1-fold). Down-regulated genes in this group include histone cluster4, h4 (*Hist4h4*, −3.6-fold), *Tgfb1* (− 2.0-fold), interleukin 16 (*Il16*, − 1.7-fold)) interleukin receptor, type 2 (*Il1r2*, − 1.6-fold), interleukin 17 receptor E (*Il17re*, − 1.6-fold) and interleukin 1 receptor accessory protein (*Il1rap*, − 2.2-fold).

Only 70 of these context-dependent genes were common to all three groups (16% of ERα^−/−^, 16% of WT-OVX and 7% of WT-SN mice). Of the genes differentially expressed in tibiae from ERα^−/−^ mice, 70 + 172 genes (55%) shared their identity with those in the WT-SN tibiae whereas only 70 + 27 (22%) were common to the WT-OVX group. A group of 51 genes were common to the WT-OVX and WT-SN groups but not those in the ERα^−/−^ group.

Of the genes differentially regulated by context compared to WT, the majority (76%) of the 172 genes common to ERα^−/−^ and WT-SN tibiae were up-regulated. Interestingly, a number of genes which were up-regulated in the tibiae from ERα^−/−^ mice were also up-regulated in those from WT-SN mice. In contrast, in comparison to the WT, of the 27 genes differentially expressed in tibiae from ERα^−/−^ and WT-OVX mice, the majority were down-regulated (ERα^−/−^ 59% and WT-OVX 78%). Of the 51 genes common to WT-OVX and WT-SN, the majority were down-regulated in both groups (69% and 65%, respectively). The majority of the 70 genes common to ERα^−/−^, WT-OVX and WT-SN tibiae were down-regulated (69%, 79% and 69%, respectively).

#### Canonical pathway analysis of genes transcriptionally regulated by context alone (WT-OVX, ERα^−/−^ and WT-SN)

Having identified the genes differentially regulated by context we used IPA software to identify canonical pathways that are significantly over-represented among them. This software utilizes a knowledge base of more than 1,000,000 functional and physical interactions for more than 23,900 mammalian genes.

Canonical pathways analysis identified 7 pathways from the Ingenuity Pathways Analysis library of canonical pathways that were most significant to the WT-OVX context (Fig. 1B). The genes for both alpha integrins (*Itga1* and *Itga4*) were down-regulated in the integrin signaling pathway. Interestingly, genes that mapped to mitochondrial dysfunction (8 genes), oxidative phosphorylation (6 genes) and glutathione metabolism (4 genes) in WT-OVX were all down-regulated in each pathway.

Significantly perturbed canonical pathways in ERα^−/−^ mice include those associated with mitochondrial dysfunction and aryl hydrocarbon receptor from the canonical signaling pathway and oxidative phosphorylation, ubiquinone biosynthesis, propanoate metabolism, glycolysis/gluconeogenesis, fatty acid metabolism and citrate cycle from the metabolic pathway. In contrast to the situation in tibiae from WT-OVX mice, 15 of the 16 genes associated with mitochondrial dysfunction and 14 of the 15 genes associated with oxidative phosphorylation pathway in the ERα^−/−^ mice were up-regulated, as were all the differentially expressed genes associated with the citrate cycle and propanoate metabolism.

In tibiae from WT-SN mice, the canonical signaling pathway which showed the most significant differential regulation was the hepatic stellate cell activation pathway (*p* = 2.0 × 10^− 5^) which involves platelet-derived growth factor (*Pdgf*), *Tgfb1*, *Il1* and tumor necrosis factor (*Tnf*) genes. Mitochondrial dysfunction (*p* =  5.69 × 10^− 3^) and oxidative phosphorylation (*p* =  1.17 × 10^− 2^) pathways were also significantly altered in WT-SN. Twelve out of 15 genes in the former and 13 out of 14 genes in the latter pathway were up-regulated.

### The effect of loading

#### The number of genes differentially regulated by loading according to context

**Fig. 2:**
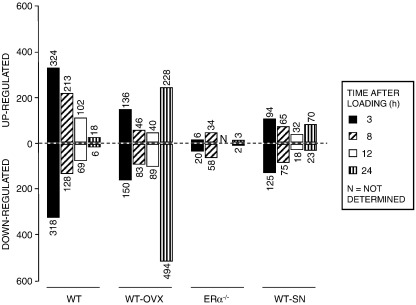
Time course of differentially expressed genes in response to loading tibiae in mice according to context. The number of genes differentially up or down-regulated at 3, 8, 12 and 24 h in tibiae from WT, WT-OVX and WT-SN mice and 3, 8 and 24 h in tibiae from ERα^−/−^ mice after a single period of *in vivo* loading compared to their contra-lateral, non-loaded controls.

**Fig. 3:**
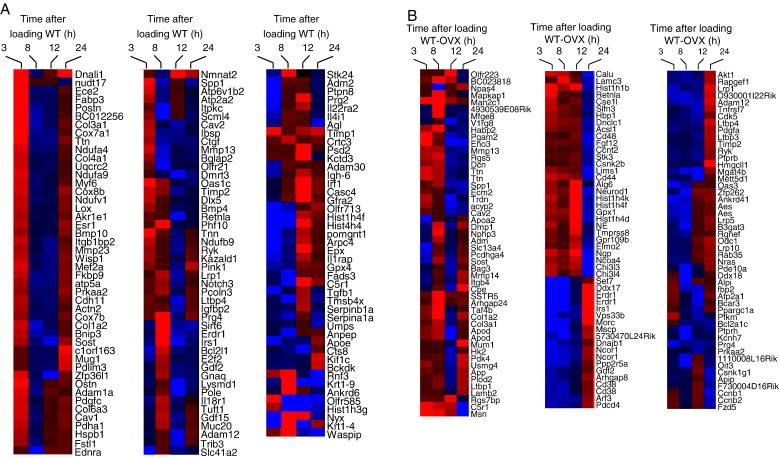

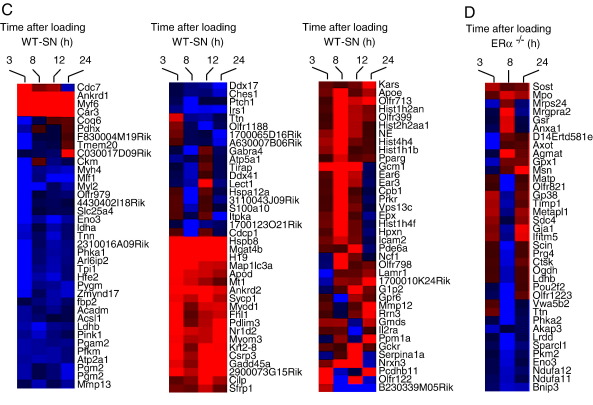
Heat maps of identified genes differentially expressed by loading according to context (A = WT, B = WT-OVX, C = WT-SN and D = ERα^–/–^). Heat map visualization of clusters formed was generated by using CLADIST. Hierarchical clustering analysis was done in Euclidean distance by average linkage method. Genes are colored based on their fold expression level. Red indicates up-regulation while blue indicates down-regulation of genes at each time point after loading. The intensity of the color indicates the level of differential regulation.

Fig. 2 illustrates the numbers of genes up- and down-regulated with loading in each context and Figs. 3A–D show the identity and a color indication of the degree of loading-related differential regulation of identified genes according to context. Complete lists of differentially regulated genes at all four time points after loading tibiae from WT mice (Table CL2) are posted at the Royal Veterinary College website (http://www.rvc.ac.uk/files/zaman2009microarraystudy).

In the tibiae of WT mice at 3, 8, 12 and 24 h after loading, there were 642 (324 up-regulated vs. 318 down-regulated), 341 (213 up-regulated vs. 128 down-regulated), 171 (102 up-regulated vs. 69 down-regulated) and 24 (18 up-regulated vs. 6 down-regulated) genes differentially regulated, respectively, compared with their non-loaded contra-lateral controls (Fig. 2). The largest impact of loading in terms of the number of genes differentially regulated was at 3 h with a gradual reduction at 8 and 12 h. In comparison, very few genes were differentially regulated by loading at 24 h. More genes were up-regulated than down-regulated at each time point after loading.

The earliest loading-related response in WT mouse tibiae involved differential up-regulation of a number of bone-related genes (osteopontin (*Spp1*, 2.0-fold), *Postn* (2.8-fold), *Ostn* (2.4-fold), *Dlx5* (1.8-fold), *Bmp4* (1.5-fold), *Bmp10* (1.5-fold), sclerostin (*Sost*, 1.6-fold), tissue inhibitor of metalloproteinase 1 (*Timp1*, 2.1-fold), *Timp2* (1.7-fold), connective tissue growth factor (*Ctgf*, 1.5-fold) and *Esr1* (1.7-fold)) (Fig. 3A, Table S4). There was also increased expression of caveolins (*Cav1*, 1.8-fold and *Cav2*, 1.6-fold) which are known to be coupled to integrin mediated mechano-transduction through the formation of focal adhesions [34,35]. A number of adhesion molecule coding genes were also up-regulated (*Tnn* (2.4-fold), *Spp1* (2.0-fold), *Postn* (2.8-fold), integrin-binding sialoprotein (*Ibsp*, 1.7-fold) and cadherin 11 (*Cdh11*, 1.6-fold)). Titin mRNA expression was up-regulated at 3 h after loading (2.1-fold) with small changes in its expression at other time points (− 1.3-, − 1.4- and 1.4-fold at 8, 12 and 24 h, respectively). Interestingly not only Timps but also MMPs genes were also up-regulated (*Mmp13* and *Mmp23* (1.8- and 1.6-fold, respectively)). *Wisp1* (1.7-fold) and platelet-derived growth factor c polypeptide (*Pdgfc*, 1.8-fold) genes were up-regulated while *Tgfb1* (− 2.1-fold) was down-regulated. Axonemal dynein light chain homolog (*Dnali1*) showed the highest expression level (11.0-fold) at 3 h in response to loading.

In WT tibiae, the expression of Wiskott–Aldrich syndrome protein interacting protein (*Waspip*), which is known to be involved in actin polymerization, was up-regulated by more than fifty-fold at 8 h after loading (Table S5). The earlier up-regulation of *Ctgf* expression was reversed at this time (− 1.6-fold), but the expression of other growth factors (*Gdf2* and *Gdf15*) as well as that of *Irs1*, *Lrp1* and tuftelin 1 (*Tuft1*) was up-regulated (1.5-, 1.7-, 2.3-, 1.6- and 1.6-fold, respectively). While the expression of the anti-apoptotic gene Bcl2-like 1 (*Bcl2l1*) gene was up-regulated (1.8-fold) at 8 h, the expression of *Bnip3* which is pro-apoptotic was down-regulated (− 1.6-fold).

In tibiae from WT mice, the most noticeable down-regulation in *Sost* expression was observed at the  12-h time point after loading (− 2.0-fold) (Fig. 3A, Table S6). This was accompanied by relatively smaller changes in its expression at 3, 8 and 24 h after loading (1.6-, − 1.3- and 1.5-fold, respectively). The expression of *Irs1*, *Ltbp4*, insulin-like growth factor binding protein 2 (*Igfbp2*) and *Cav2* was also down-regulated (− 2.6, − 1.9, − 1.6 and − 1.6-fold, respectively) (Table S6). *Ttn* (2.1-fold), *Timp1* (1.8-fold) and *Sost* (1.5-fold) were among the few genes that were differentially regulated at 24 h after loading WT mice (Fig. 3A, Table S7).

In the tibiae of WT-OVX mice, the number of genes differentially regulated by loading at 3, 8 and 12 h was lower than in the WT at the equivalent time points (286 vs. 642, 129 vs. 341 and 129 vs. 171, respectively) (Fig. 2). Whereas the differential response in the tibiae of WT had reduced substantially by 24 h (to 24 genes) in WT-OVX, this was the time of greatest differential response (722 genes). Furthermore, in contrast to the response in WT tibia, the differentially regulated genes in the WT-OVX tibiae were predominantly down-regulated at each time point. Complete lists of differentially regulated genes at all four time points after loading tibiae from WT-OVX mice are posted (Table CL3) at the Royal Veterinary College website (http://www.rvc.ac.uk/files/zaman2009microarraystudy).

In the tibiae from WT-OVX mice, loading-induced suppression of *Sost* expression was achieved at an earlier time point in comparison to the WT (− 1.7-fold at 3 h) (Fig. 3B, Table S8). This situation was then reversed at 8 h (1.5-fold) and the pattern of *Sost* expression at 12 and 24 h was similar to that seen in WT mice after loading (− 1.5- and 1.3-fold, respectively). *Irs1*, *Lrp1*, *Gdf2* and cluster of differentiation 38 (*Cd38*) were also down-regulated (− 3.5, − 1.8, − 1.6 and − 1.6-fold, respectively). In common with the situation in the tibiae of WT mice, *Ttn* expression in the tibiae of WT-OVX mice was up-regulated 1.8-fold at 3 h after loading.

*Irs1* and *Cd38* expression continued to be suppressed at 8 h after loading in tibiae from WT-OVX mice (− 1.7-fold) while the suppression of *Ttn* was de-repressed resulting in higher expression of this gene (Table S9). At 12 h, the expression of Dentin matrix protein 1 (*Dmp1*) expression was up-regulated (1.9-fold) while the expression of a number of other genes was down-regulated in tibiae from these WT-OVX mice including (*Cav2,* − 1.7-fold) breast cancer anti-estrogen resistance 3 (*Bcar3,* − 1.9-fold), tumor necrosis factor receptor superfamily member 7 (*Tnfrsf7*, − 1.7-fold), *Bcl2* associated athanogene 3 (*Bag3*, − 1.7-fold) and *Ppargc1a* (− 1.6-fold) (Fig. 3B, Table S10).

In tibiae from WT-OVX mice, the largest number of genes differentially regulated in response to loading was at 24 h. The number of down-regulated genes at this time was twice the number up-regulated. The down-regulated genes included a number involved in adhesion (Lim and senescent cell antigen-like domains 1 (*Lims1*, − 2.1-fold), *Cd44* (− 1.9-fold), *Spp1* (− 1.7-fold)), Wnt signaling pathways (frizzled 5 (*Fzd5*, − 1.6-fold), casein kinase II beta subunit (*Csnk1g*, − 1.8-fold) and *Csnk2b* (− 1.5-fold)) and matrix mineralization extracellular matrix protein 2 (*Ecm2*, − 1.6-fold) and *Mmp13* (− 1.5-fold) (Fig. 3B, Table S11). However, both *Lrp1* (1.9-fold) and *Lrp10* (1.5-fold) as well as a number of genes related to TGFβ (latent transforming growth factor beta binding protein 1 (*Ltbp1*, 1.6-fold), *Ltbp3* (1.6-fold) and *Ltbp4* (1.8-fold)) were up-regulated.

The time of greatest differential response to loading in tibiae of WT-SN mice was at 3 h which is similar to that observed in WT mice (Fig. 2). In common with WT-OVX mice, loading-related differential gene expression in tibiae of WT-SN mice was always lower than in WT at each time point with the exception of 24 h after loading. At this time point, in common with the WT-OVX, the number of genes differentially regulated by loading was larger than in tibiae of WT. The up- and down-regulated gene expression pattern in response to loading in tibiae of WT-SN mice was similar to that observed in WT-OVX at the earlier time points (3 and 8 h) and to WT at the later time points after loading (12 and 24 h). Complete lists of differentially regulated genes at all four time points after loading tibiae from WT-SN mice are posted (Table CL4) at the Royal Veterinary College website (http://www.rvc.ac.uk/files/zaman2009microarraystudy).

In common with tibiae from WT and WT-OVX mice, in those from WT-SN mice the largest change in loading-induced *Ttn* expression was at 3 h (1.8-fold) (Fig. 3C, Table S12). Cell division cycle 7 (*Cdc7*) gene expression was up-regulated 27-fold at this time, while the expression of *Myf6* was markedly up-regulated in all four time points after loading in tibiae from WT-SN mice (4.3-, 5.1-, 4.7- and 4.5-fold) (Fig. 3C, Table S12-S15). The expression of *Irs1* was down-regulated in all four sampling times after loading in these mice (− 1.4, − 2.6, − 1.9 and − 2.3-fold). *Sost* expression levels were not altered in response to loading in tibiae from WT-SN mice at any of the four sampling time points. Neurectomy seemed to be associated with up-regulation of a large number of genes related to adhesion. Loading these mice did not down-regulate the expression levels of these genes. On the contrary, the expression level of one adhesion gene, Protocadherin beta 11 (*Pcdhb11*), was strongly up-regulated at 24 h after loading (21-fold).

Perhaps the most obvious effect of context on the number of genes regulated by loading was that in the tibiae of ERα^−/−^ mice. In this group, the number of genes differentially regulated by loading was far less than in any of the other groups, 26 vs. WT 642 at 3 h, a maximum of 92 vs. WT 341 at 8 h and 5 vs. WT 24 at 24 h (Fig. 2). Complete lists of differentially regulated genes at all three time points after loading tibiae from ERα^−/−^ mice are posted (Table CL5) at the Royal Veterinary College website (http://www.rvc.ac.uk/files/zaman2009microarraystudy).

In common with tibiae from WT, in the ERα^−/−^ mice *Sost* was up-regulated (1.6-fold) at 3 h after loading (Fig. 3D, Table S16). However, unlike WT, in ERα^−/−^ mice there was no subsequent down-regulation of *Sost* at the later time points analyzed (8 or 24 h) (Fig. 3D, Tables S17 and S18). Apart from 2.2-fold down-regulation at 8 h, *Ttn* expression levels did not change markedly at 3 and 24 h after loading (1.3 and − 1.1-fold, respectively).

#### Loading-related differential expression of genes which are common to loading in WT according to context

**Fig. 4:**
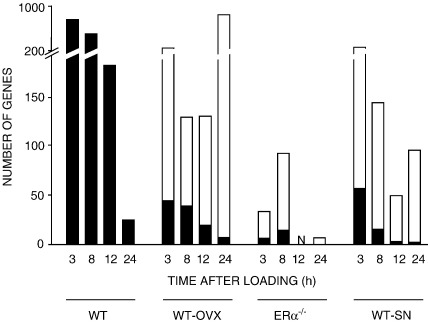
The number of genes at each time point, in each context, that are differentially regulated by loading that are common to the loading response in tibiae from WT mice. Loading-induced differentially expressed genes in tibiae from WT-OVX, ERα^−/−^ and WT-SN mice are separated into those common to (black) and different from (white), those regulated in the tibiae from WT mice at the equivalent time point after loading.

The analysis in Fig. 4 separates those genes differentially expressed by loading into genes common to and different from those differentially expressed in response to loading in the WT. Only 8% of the genes differentially transcribed at 3, 8, 12 and 24 h after loading in tibiae of WT-OVX animals were homologous to the differentially transcribed genes at the equivalent time point after loading in WT mice. At 3 h after loading 15% of WT-OVX (44/286), 23% of ERα^−/−^ (6/26) and 25% of WT-SN (55/219) respectively were homologous to the differentially transcribed genes in WT animals at the same time point.

#### Validation of microarray data

**Fig. 5:**
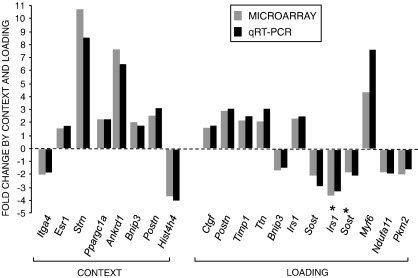
Comparison of the fold change in regulation of 16 sample genes as assessed by the microarray and quantitative real-time RT-PCR with β-actin as an internal control. Target genes were selected based on interest and fold change in the microarray both in up- and down-regulation according to context or in response to loading in different contexts. Genes differentially expressed by context were selected from WT-OVX mice vs. WT mice tibiae (*Itga4*, *Esr1*), ERα^−/−^ mice vs. WT mice tibiae (*Ppargc1a* and *Strn*) and WT-SN mice vs. WT mice tibiae (*Ankrd1*, *Bnip3*, *Postn* and *Hist4h4*). Genes differentially expressed by loading in WT mice tibiae were selected from 3 h (*Ctgf*, *Postn*, *Timp* and *Ttn*), 8 h (*Bnip3* and *Irs1*) and 12 h time points (*Sost*). Genes differentially expressed by loading in WT-OVX mice tibiae were selected from 3 h time point (*Irs1*⁎ and *Sost*⁎). Gene differentially expressed by loading in WT-SN mice tibiae was selected from 3 h time point (*Myf6*). Genes differentially expressed by loading in ERα^−/−^ mice tibiae were selected from 8 h time point (*Ndufa11* and *Pkm2*).

Validation of microarray data was carried out using quantitative real-time RT-PCR on 16 target genes with β-actin as an internal control. Target genes were selected based on interest and fold change both in up- and down-regulation according to context or in response to loading in different contexts. In all cases, the results from qRT-PCR supported the findings from the microarray (Fig. 5).

#### Functional analysis of genes differentially regulated by loading according to context

**Fig. 6:**
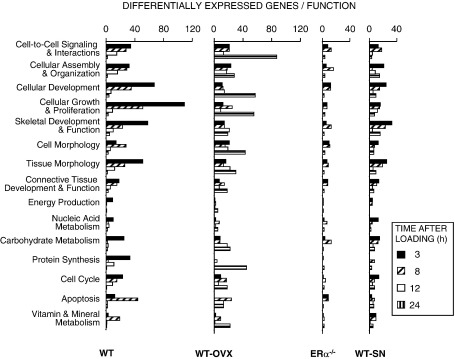
Functional analysis of genes differentially expressed by loading in tibiae from WT, WT-OVX, ERα^−/−^ and WT-SN mice mapped into 1 of 15 functional groups using the Ingenuity Pathway Analysis knowledge data base. The number of “focus” genes in each of these 15 functional categories that were most significantly over-represented (10^− 5^ < *p* > 10^− 2^) are shown for all four contexts.

Since identification of individual genes conveys little indication of function, we compiled a list of genes differentially regulated according to their role as part of complexes, pathways and biological networks using the Ingenuity systems functional analysis. This analysis identifies the biological functions that are most significant to the differentially regulated genes. Fisher's exact test was used to calculate a *p*-value determining the possibility that each biological function is due to change alone. Fig. 6 shows the 15 functional categories that are most significantly over-represented (10^− 6^ < *p* > 10^− 2^ by Fischer's exact test) in the list of genes differentially regulated in response to loading and the number of genes in each category differently expressed in WT mouse tibiae at 3 h after loading. These chosen functions were then used as a template to analyze the changes in gene expression in loaded tibiae from WT-OVX, ERα^−/−^ and WT-SN mice at all sampling points after loading.

In the tibiae of WT mice, with the exception of the functional category “Apoptosis and Vitamin and Mineral Metabolism”, the maximum change in gene expression in each functional category occurs at 3 h after loading, and by 24 h the pattern of differential expression has been reduced to almost nothing in all functional groups. The loaded tibiae from WT-OVX mice show a similar level of differential gene expression to that seen in the WT but the highest level of differential expression occurs later; little change occurring at 3 h and a substantial change occurring at 24 h. In common with the WT, the pattern of loading-induced differential gene expression in each function in WT-SN tibiae was most pronounced at the 3 -h time point after which it also declined in a similar manner to that seen in the WT. However, the most prominent feature of these data is the low level of differential gene expression in each of these functional categories in the loaded tibiae of ERα^−/−^ mice.

#### Canonical pathway analysis of genes transcriptionally regulated by loading according to context

**Fig. 7:**
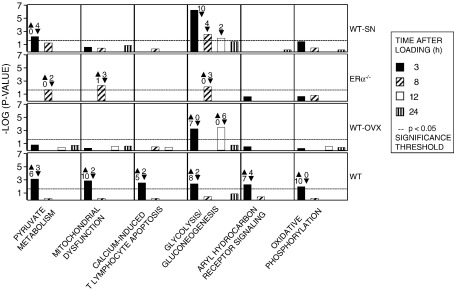
Canonical pathway analysis of genes differentially regulated by loading mouse tibiae according to context. Canonical pathways that were most significant to the focus genes differentially regulated at the earliest sampling time (3 h) after loading of WT mouse tibiae were constructed using the IPA knowledge data base. Using this set of canonical pathways as a template, focus genes differentially regulated by loading at all sampling points in all four contexts were processed to find the degree to which these pathways were represented in response to loading in each group of mice. A negative log value of 1.3 has only a 5% chance of being generated by chance alone.

In tibiae from WT mice, the canonical pathways that showed the most significant differential regulation in response to loading at the earliest sampling time (3 h) were in the pathways of pyruvate metabolism, mitochondrial dysfunction, calcium-induced T lymphocyte apoptosis, glycolysis/gluconeogenesis, aryl hydrocarbon receptor signaling and oxidative phosphorylation. The level of significance of differential regulation of each of these pathways was reduced at the later sampling points. Using this set of canonical pathways as a template, genes differentially regulated by loading at all sampling time points in tibiae from WT, WT-OVX, ERα^−/−^ and WT-SN mice were processed using IPA (Fig. 7).

Differential regulation of the pathways involved in oxidative phosphorylation, aryl hydrocarbon receptor signaling and calcium-induced T cell apoptosis reached significance only in WT loaded mice. The pattern of response in tibiae from WT-OVX mice differed from that in WT mice with only the glycolysis/gluconeogenesis pathway reaching significance at 3 and 12 h after loading. In ERα^−/−^ mice, none of the canonical pathways reached significance at 3 h after loading and only pyruvate metabolism, mitochondrial dysfunction and glycolysis/gluconeogenesis pathways reached significance at 8 h. In common with WT and in contrast with WT-OVX and ERα^−/−^, WT-SN loaded tibiae achieved significance in both the pyruvate metabolism and glycolysis/gluconeogenesis pathways at the earliest sampling point after loading. In tibiae from WT-SN mice, changes in the glycolysis/gluconeogenesis pathway reached significance at 8 and 12 h after loading.

Although establishing which canonical pathways show significant responses to loading in different contexts is useful, it is probably more informative to look at the differential regulation of genes which precipitate perturbations in these pathways. Mitochondrial dysfunction is significantly regulated in response to loading in both WT and ERα^−/−^ tibiae but the commonality ends there. In tibiae from WT mice the genes associated with this pathway, coding for products which catalyse the transfer of electrons in the inner mitochondrial membrane, are all up-regulated (NADH dehydrogenase 1 alpha subcomplex 4 (*Ndufa4*, 1.9-fold), *Ndufa9* (1.9-fold), *Ndufb9* (1.7-fold), cytochrome C oxidase subunit VIIa polypeptide 1 (*Cox7a1*, 2.7-fold), *Cox7b* (1.5-fold), *Cox8b* (1.8-fold) and ATP synthase subunit alpha (*Atp5a1*, 1.6-fold)). In contrast, in tibiae from ERα^−/−^ mice loading down-regulated the expression of electron transport-related genes (*Ndufa11* (− 1.7-fold), *Ndufa12* (− 1.7-fold) and oxoglutarate dehydrogenase (*Ogdh*, − 1.7-fold). Thus, while a single short period of loading the tibiae of WT mice initiates a cascade of gene transcription in preparation for increasing utilization of cellular energy, this is not the case in tibiae from ERα^−/−^ mice. In tibiae from WT mice, 9 of the genes related to pyruvate metabolism and glycolysis/gluconeogenesis were up-regulated. These were (acylphosphatase 2 (*Acyp2*, 1.6-fold), aldehyde dehydrogenase 9 family member A1 (*Aldh9a1*, 1.6-fold)), dihydrolipamide 5-acetyltransferase (*Dlat*, 1.5-fold), fructose-1,6-biphosphatase 2 (*Fbp2*, 1.9-fold), lactate dehydrogenase B (*Ldhb*, 2.1-fold), malate dehydrogenase 1 (*Mdh1*, 2.3-fold), pyruvate dehydrogenase alpha 1 (*Pdha1*, 1.7-fold), phosphoglucomutase 3 (*Pgm3*, 2.1-fold) and triosphosphate isomerise 1 (*Tpi1*, 1.5-fold). Three genes were down-regulated (*Acyp1*, − 1.7-fold), hydroxyacylglutathione hydrolase (*Hagh*, − 2.7-fold) and *Ldhc* (− 1.7-fold) in response to loading. The number of genes which were up or down-regulated in the canonical pathways that reached significance in response to loading at each time point in each background is shown in Fig. 7.

The pathways shown in Fig. 7 are based on the template generated by using the differentially regulated genes in response to loading WT mice at 3 h after treatment. Tibiae from mice with different background and sampled at different time points after loading generated their own significant canonical pathways (*p* <  0.05 using the right-tailed Fisher's exact test). These significant pathways for each background and each time point are shown in Table 2.

## Discussion

One of the remarkable features of bones' response to mechanical loading is the short duration of loading required to modify bone modeling and remodeling [22,36-41]. In the experiments reported here, a single 30-s period of dynamic loading engendering strains of physiological magnitude was sufficient to initiate a cascade of events culminating days later in measurable increase in bone formation. One experimental advantage of this phenomenon is that it is possible to follow the time course of events involved in the post-loading cascade following a single stimulatory event. The shortness of the stimulatory “pulse” is significant in this context since it avoids the problem of trying to identify a single sequence of post-loading events in a system which has been exposed to and is responding to, either a single prolonged period of stimulation or a series of separate periods of stimulation.

Previously documented efforts to determine the earliest cell changes involved in the transduction of strain-related events into cellular responses have identified almost immediate fluxes in calcium both within the cell and between the cell and its extra-cellular environment [42,43]; rearrangements in the cells' cytoskeleton and their attachments to the surrounding matrix [44,45]; the production of prostanoids and nitric oxide (NO) [46-49]; and activation of ERK signaling [11,12]. These changes occur within minutes of exposure to the strain-related stimulus. Our study aimed to establish the pattern of gene expression using microarray analysis in the 24 h following a single loading-related osteogenic stimulus in four different contexts each with established relevance to the loading-related response. The contexts were normality (WT), estrogen deficiency (WT-OVX), disuse (WT-SN) and the absence of ERα (ERα^−/−^). Each of the different contexts was associated with a distinct pattern of gene expression in which hundreds of genes were differentially expressed. The effects of loading in these different contexts were similarly associated with differential expression in hundreds of genes.

In none of the contexts we examined was there marked differential regulation of the genes directly associated with the immediate signaling events just referred to (prostaglandins, NO and ERK) except those related to integrin signaling and caveolin. These immediate signaling events primarily involve changes in the activity of factors already present rather than their production through transcriptional regulation of the relevant genes. Regulation of production presumably occurs over a longer time period and in most cases follows, rather than is required for, changes in the activity of cell signaling pathways. In these experiments, our earliest time point after loading (3 h) was chosen to catch the earliest changes we expected in gene regulation. The large differences in gene expression that we observed at this time point suggest that this regulatory response was well under way by this time and that an earlier time point would have been desirable.

Estrogen deficiency is known to cause significant bone loss in mice as well as in humans [50,51]. In our present study, lack of estrogen associated with ovariectomy (WT-OVX) was associated with an increase in expression levels of mRNA for ERα, an estrogen receptor subtype that plays an important role in bone biology. This is in agreement with previous findings where a reduction in estrogen levels brought on by OVX in rats [33] and menopause in women [52] resulted in marked increases in ERα mRNA levels. This may be a compensation mechanism as we [18] and others [33] have reported a significant decline in ERα protein levels in response to ovariectomy.

Seven pathways significant to the WT-OVX context were identified from the Ingenuity Pathways Analysis library. The most significant of these was the integrin signaling pathway. Importantly the expression of alpha integrins associated with this pathway was down-regulated by OVX thereby possibly compromising integrin mediated signaling. Genes that mapped to the mitochondrial dysfunction and oxidative phosphorylation pathways were all also down-regulated in WT-OVX. This is particularly interesting in relation to the recently reported relationship between bone and energy metabolism [53].

We previously reported not only that the osteogenic response to loading was smaller in Korach-derived ERα^−/−^ mice than WT [14] but that the osteoblast-like cells derived from these mice had a higher basal level of NO which was not elevated further by mechanical strain [54]. At the time that we made this observation we could offer no explanation for it. Data from the current study suggest a possible mechanism. The Korach-derived ERα^−/−^ mice express a short (46 kDa) form of ER produced by alternative splicing [55] which, it has been suggested, may trigger rapid, non-genomic signal responses [56]. Our microarray data analysis shows that the basal expression of *Strn*, the gene for striatin is up-regulated by 10.7-fold in ERα^−/−^ mice. Striatin acts as a molecular anchor that localizes ERα to the membrane caveolae and organizes an ERα-eNOS membrane signaling complex for a rapid, non-genomic activation of eNOS [57]. Interestingly, expression levels of *Cav1*, *Cav2* and *Cav3* are also up-regulated in ERα^−/−^ mice. Elevated expression levels of *Strn* and caveolins may be responsible for the higher basal levels of NO reported in osteoblast-like cells derived from ERα^−/−^ mice, and this in turn may be associated with a higher trabecular bone volume and bone mineral density reported in these mice [58].

We have previously reported that mechanical loading *in vivo* caused a small but positive effect on the expression of ERα protein on the medial surface of the ulna where loading stimulates reversal from bone resorption to formation [18]. Consistent with this finding, there was a small loading-related increase in the expression of *Esr1* mRNA expression in tibiae from WT mice.

A possible relationship between the level of expression of genes associated with oxidative phosphorylation and the bone mass observed in WT-OVX and ERα^−/−^ mice does not hold true in the case of WT-SN mice. Here, neurectomy-induced bone loss occurs on a background of increased expression of genes associated with mitochondrial dysfunction and oxidative phosphorylation pathways. Other studies have also implicated canonical Wnt/β-catenin signaling in the regulation of bone mass [59,60]. Eight genes associated with Wnt/β-catenin signaling were up-regulated in WT-SN mice, four of which code for known inhibitors of the Wnt/β-catenin pathway (*Sfrp1*, *Sfrp4*, *Sfrp4* and *Dkk3*). Reduced signaling through the Wnt/β-catenin pathway could be responsible for the reduced bone mass in WT-SN mice [24]. Possibly as a compensatory mechanism in tibiae from WT-SN mice, the expression of a number of growth factor genes was up-regulated. A large number of genes involved in adhesion were also up-regulated in tibiae from WT-SN mice. Although sciatic neurectomy is a widely used model of disuse [61], it is known to induce some degree of physiological stress, although probably less than tail suspension, as well as reduced innervation of the neurectomized limb [62]. It is possible therefore that the neurectomy-induced changes in bone modeling/remodeling may not be due to unloading alone.

Comparison of the gene expression profile of the loaded and non-loaded tibiae in WT mice revealed differential up-regulation of a number of bone-specific genes. One of the genes markedly up-regulated at 3 h after loading was osteopontin. Previous analysis of the osteopontin gene revealed a mechanical stress response element(s) within its promoter region [63]. The expression levels of this gene are known to be mechanically responsive in osteocytes *in vivo* [64]. Although osteopontin is generally considered as a structural bone protein linking bone cells to the bone extracellular matrix, it has also been reported to stimulate cell activity through multiple receptors linked to several signaling pathways that relate to its function in the formation and remodeling of bone [65]. In contrast to WT mice, osteopontin expression levels were only down-regulated by loading in tibiae from WT-OVX mice and remained unchanged in tibiae from WT-SN and ERα^−/−^ mice at all time points after loading.

Periostin is an osteoblast-specific factor which has a role in cell adhesion. Although it has not previously been implicated with bone loading, *Postn* expression was up-regulated by loading in this study. It has recently been reported that a periostin-like factor stimulated bone formation *in vivo* possibly by recruitment and attachment of osteoblast precursors [66] as well as by promoting osteoblast proliferation and differentiation [67]. Periostin knockout mice have low levels of trabecular bone [68]. Osteocrin expression was also similarly up-regulated by loading. It is a bone-specific protein secreted by RUNX2-positive osteoblasts and newly formed osteocytes that modulate the osteoblast phenotype [69]. Its expression has been reported in human osteoblasts at sites of bone remodeling [70]. Neither *Postn* nor *Ostn* expression levels were modulated by loading in any of the contexts we examined except WT.

Expression levels of *Sost,* the gene for sclerostin, were modulated by loading in tibiae from WT, WT-OVX and ERα^−/−^ mice but not those from WT-SN. In tibiae from WT and ERα^−/−^ mice, *Sost* expression levels were elevated to a similar extent at 3 h after loading. This early up-regulation was reversed only in WT mice leading to a 2-fold down-regulation whereas in ERα^−/−^its levels remained above that of the non-loaded controls. In contrast to WT and ERα^−/−^ mice, *Sost* expression was down-regulated in WT-OVX mice earlier after loading (3 h). In common with WT, in WT-OVX *Sost* expression was also down-regulated at 12 h after loading. This down-regulation of *Sost* by loading is in agreement with previous reports where expression of sclerostin was down-regulated by loading and up-regulated by unloading [71-73]. The Wnt/β-catenin signaling pathway is known to be important in regulating bone mass [74]. It has recently been proposed that sclerostin, a protein which is almost osteocyte specific, plays an essential role in mediating bone's response to loading through antagonizing Wnt/β-catenin signaling [75]. Thus, lower levels of *Sost*/sclerostin expression would lead to enhanced Wnt/β-catenin signaling resulting in a load-related osteogenic response. The lack of down-regulation of *Sost* expression in tibiae from ERα^−/−^ mice indicates disruption in this potentially critical strain-related pathway.

In addition to the “usual suspects”, the microarray analysis identified titin as one of the genes up-regulated in response to loading. Titin is predominantly expressed in muscle where it is involved with sarcomere assembly and more recently it has been implicated in strain sensing in muscle cells [76]. In bone cells, titin expression has been reported in monolayer cultures of MG-63, osteoblast-like cells [77]. More recently, an *in vivo* study has shown that titin mRNA expression is 2.1-fold higher in osteocytes in comparison to osteoblasts in a 5- to 8-day-old mouse calvaria [78].

*Dnali1,* the gene coding for axonemal dynein light chain homolog, showed the highest expression level at 3 h in response to loading (11-fold) in tibiae from WT mice. However, its expression levels did not change with loading in any of the other contexts. *Dnali1* is predominantly expressed in the testis but more recently its expression has been described in other tissues (brain, ovary, kidney and lung) [79]. The precise function of this protein is not known; however, it is a potential candidate for immotile cilia syndrome [80].

One of the most significant features of this microarray analysis is the loading-related differential regulation of bioenergetic pathways. It has recently been reported that in response to osteogenic stimuli, osteoblasts switch over to oxidative phosphorylation for energy generation [81]. Major contributors to oxidative phosphorylation are the five complexes of the respiratory chain located on the inner mitochondrial membrane. Canonical pathway analysis revealed that in WT tibiae 10 genes linked to complex I, III, IV and V of the oxidative phosphorylation pathway were up-regulated in response to loading. Load also up-regulated 8 out of 10 genes associated with the glycolysis pathway. Thus, the pathways involved in generating cellular energy are up-regulated in response to loading in the WT and WT-OVX context. Genes associated with Glycolysis were down-regulated with loading in tibiae from ERα^−/−^ and WT-SN mice. In common with WT, pyruvate metabolism pathways associated genes were down-regulated by loading after 3 h in WT-SN mice.

We and others have previously shown that mechanical strain induces proliferation of osteoblast-like cells [82,83]. Cellular proliferation is governed by the cell cycle which in turn is controlled by the relative levels of individual cyclin family members. Cyclin E2 is known to promote the cell cycle progression through G1/S phase transition [84]. Consistent with this, cyclin E2 expression was up-regulated in response to loading. The defining feature of S phase is replication of the genome. Histones constitute half the mass of chromatin and play a crucial role in packaging of DNA required for this phase. The synchronized biosynthesis of histones is also regulated by cyclin E2-CDK2 [85]. Consistent with this finding the transcription of mammalian core histone gene, H4, was up-regulated by loading in WT mouse tibia.

In addition to activating signaling pathways leading to proliferation, pro-apoptotic gene expression was down-regulated and anti-apoptotic gene expression was up-regulated at 8 h after loading in tibiae from WT mice. This triple pronged response to a pulse of loading would result in a higher number of osteoblasts per bone surface leading load-induced osteogenesis.

With the exception of *Sost*, which was down-regulated at an earlier time point than in the WT, loading the tibiae of WT-OVX mice did not differentially regulate the expression of the bone-related genes (*Spp1*, *Ostn*, *Postn*, *Timp1*, *Timp2* and *Ctgf*) detected in the loaded WT mice. Consistent with this lack of early response to loading, the expression of osteopontin, *Irs1*, *Gdf2*, *Lrp1*, *Lrp5* and *Cd38* was down-regulated. CD38^−/−^ mice are known to display a marked reduction in bone density [86]. This divergence in loading-related response was further highlighted by the finding that in contrast to the WT, in the WT-OVX loading did not induce an up-regulation of the genes related to the Glycolysis pathway. On the contrary, they were down-regulated by loading. Although these data argue for a blunted osteogenic response to loading in WT-OVX, this is in contrast to the study of Hagino et al. [87], which showed that ovariectomy did not change bones' response to loading.

To the best of our knowledge, there are no other reports of a microarray analysis after a brief bone loading session *in vivo* such as that we report here. The most similar study is that reported by Xing et al. [88] who used microarray to analyze global gene expression in response to a number of periods of loading of the mouse tibia in four-point bone bending. These authors extracted total RNA from the mouse tibiae 24 h after the last loading period. Forty-four of the genes differentially regulated by loading in all 4 backgrounds in our study were also reported to be differentially regulated by Xing et al. Twenty-three of these genes were homologues to the genes differentially regulated over 24 h in WT loaded tibia in our study. These were (procollagen-lysine (*Plod2*), pyruvate dehydrogenase, isozyme 4 (*Pdk4*), *Timp4*, ADP-ribosylation factor-like 6 (*Arl6*), receptor-like tyrosine kinase (*Ryk*), mannose receptor, C type 2 (*Mrc2*), PTPRF interacting protein-binding protein 1 (*Ppfibp1*), lysyl oxidase (*lox*), *Mmp23*, sycoglycan beta (*Sgcb*), *Tnn*, *Timp1*, integral membrane protein 2A (*Itm2a*), FK506-binding protein 9 (*Fkbp9*), vesicle docking protein (*Vdp*), gap junction membrane protein alpha 1 (*Gja1*), cathepsin D (*Ctsd*), AT motif-binding factor (*MAtbf1*), integrin-binding sialoprotein (*Ibsp*), matrilin 2 (*Matn2*), lipase (*Lipc*), *6330577ERik* and *2610024B07Rik*). In common with our study, none of the candidate genes whose expression has been shown in other studies to be regulated by mechanical loading were differentially regulated to a significant level in the study of Xing *et al*.

Recently, Zhang et al. [89] performed a microarray analysis where joint loading was used to drive bone formation in mouse tibiae. In common with Xing et al., multiple loading sessions were used over three consecutive days and RNA extracted 1 h after the last loading session. Seven of the 25 genes significantly up-regulated in this study were also up-regulated in WT loaded tibiae in our study. These were mas-related gpr (*Mrgpr*), tissue inhibitor of metalloproteinase 1 (*Timp1*), thrombospondin (*Thbs*), procollagen type III alpha1 (*Col3a1*), mast cell protease (*Mmcp*), matrilin 2 (*Matn2*) and proteoglycan 4 (*Prg4*). Since only some of the genes differentially regulated by loading in this study were listed in their manuscript (50 out of 441 genes), it is difficult to make an exact comparison between the studies. However, signaling pathways generated in this study when all the 441 loading-related genes were accounted for showed an overlap with our current study. ECM remodelling, TGFβ and Wnt signaling pathways were differentially regulated in both studies. Differences in the loading response among the three studies may partly be due to the three different types of loading regimens used (axial, four-point bending and joint loading as well as single or multiple loading sessions), different sampling compartments of bone used for RNA extraction (cortical only or cortical and trabecular combined) and different sampling time points after the last loading session.

In summary, a single short period of *in vivo* bone loading, which stimulates an osteogenic response, results in up-regulation and down-regulation in the levels of expression of hundreds of genes within the bones' resident cells over the period from 3 to 24 h after loading. The number and identity of the genes regulated by loading and the timing of their regulation are substantially influenced by context (normal, estrogen deficient, lack of ERα or disuse). Many of the genes differentially regulated within this early loading-related response are not specific to bone but are primarily involved in energy metabolism, cell adhesion and proliferation. In none of the contexts examined were there any loading-induced changes in expression of the iconic candidate mechano-sensitive genes such as IGFs, COX2 and NOS. In the situation of estrogen deficiency caused by ovariectomy and lack of normal functional loading following sciatic neurectomy, artificial loading-induced changes in the expression of genes involved in canonical signaling pathways were generally delayed compared with those in similarly loaded tibiae in WT mice. In mice lacking ERα the loading-related response in terms of gene regulation at all time points was markedly smaller than in any of the other contexts investigated.

## Figures and Tables

**Table 1:** Quantitative real-time RT-PCR primer sequences (5′ → 3′).

Gene	Sequence (forward)	Sequence (reverse)	Position	Length (bp)	Accession no.
*Ankrd1*	TGAATGAAGCAACAGAAGG	ACCAAACCGAACCAAATC	1451–1557	107	NM_014468
*Bnip3*	TCATTAAAGGGTTTTCCCCAAAGG	GCTTCTACTTGCTACTCAGTTCAC	1080–1207	128	NM_009760
*Ctgf*	CAAACTCCAAACACCATAGG	AATCTGACTTCCAATACATAGC	2034–2178	145	NM_010217
*Esr1*	ACCATTGACAAGAACCGGAG	CCTGAAGCACCCATTTCATT	875–1044	170	NM_007956
*Hist4h4*	CATCTCGGGTCTCATCTAC	ATAGCCGTAACCGTCTTG	138–254	117	NM_175652
*Irs1*	GTTCATAATACTAGACACTGTTGG	CTTCTCGGAGGCAATGTATG	5666–5787	122	NM_010570
*Itga4*	CAGACAACTACAGGGCAATG	GCAATACAGGAGTCTCTTATCAG	2771–2905	135	NM_010576
*Myf6*	CTCAGCCTCCAGCAGTCTTC	GTTACTTCTCCACCACCTCCTC	655–755	101	NM_008657
*Ndufa11*	CCTCTTGCCTCCATCTACC	ACCTAGCCTAGTTCCACATAG	2824–2939	116	BC053075
*Pkm2*	AACGCTTGTAGTGCTCAC	AGTCCTGCATTCCTCCTC	1719–1864	146	NM_011099
*Postn*	ACCCAAAGCACACAGTTACC	AATTTAGCAGGAAACCCACATTG	2410–2522	130	NM_008904
*Ppargc1a*	ATTCCACCAAGAGCAAGTAT	CGCTGTCCCATGAGGTATT	2816–2956	141	NM_015784
*Sost*	GAGAGAGCGTTTGTAACAGAAG	GGCTTTCAGTCTTTGTGGATG	1773–1878	106	NM_024449
*Strn*	GACGGCACTCTTCGCTTATG	CCTTGCTGAACGATGCTACC	1841–1982	142	NM_011500
*Timp1*	CATCCTCTTGTTGCTATCAC	GCTGGTATAAGGTGGTCTC	197–345	149	NM_011593
*Ttn*	ACTGGATGTGGCTGATGTC	CGATGTAGTTGGTGACCTTG	93697–93715	128	NM_011652
*Actb*	CTATGAGCTGCCTGACGGTC	AGTTTCATGGATGCCACAGG	798–893	114	BC063166

**Table 2:** Canonical signaling pathways significantly perturbed in response to loading WT, WT-OVX, ERα^−/−^ and WT-SN mouse tibia.

WT	OVX	ERα^−/−^	SN
3 h			
Pyruvate metabolism	Glycolysis/gluconeogenesis	Caveloar-mediated endocytosis	Glycolysis/gluconeogenesis
Mitochondria dysfunction	Propanoate metabolism	Hepatic stellate cell adhesion	Propanoate metabolism
Calcium-induced apoptosis		Ephrin receptor	Calcium
Glycolysis/gluconeogenesis		Leukocyte extravasation	Pyruvate metabolism
Aryl hydrocarbon receptor			Fructose mannose metabolism
Oxidative phosphorylation			Actin cytoskeleton
			Pentose phosphate pathway

8 h			
Ephrin receptor	Nitric oxide	Mitochondrial dysfunction	Calcium
FMLP	Ceramide	Glycolysis/gluconeogenesis	Glycolysis/gluconeogenesis
Interferon	VDR activation		Hepatic stellate cell activation
Integrin	CTLA4		
CXCR4	IGF-I		
CCR3	α-Adrenergic		
Actin cytoskeleton	NFAT		
Huntington's disease	Glucocorticoid receptor		
IL8	FcyRIIB		
	Estrogen receptor		

12 h			
Complement system	Glycolysis/gluconeogenesis	N	Glycolysis/gluconeogenesis
	Regulation of actin-based mobility by Rho		Inosital metabolism
	Fructose and mannose metabolism		
	Glucose metabolism		

24 h			
Hepatic stellate cell activation	IL-4	EGF	Calcium
Pentose phosphate pathway	Protein ubiquitination pathway	Caveolar-mediated endocytosis	Hepatic stellate cell activation
	Fatty acid elongation in mitochondria		Tight junction
	NFAT		
	IL-10		
	VEGF		

N = Not determined.

## Appendix A

Supplementary material

Table CL1 Fold change of differentially expressed genes in WT-OVX mice vs. WT mice.

Table CL2 Fold change of differentially expressed genes in WT mice at 24h after loading compared with their non-loaded contra-lateral controls.

Table CL3 Fold change of differentially expressed genes in WT-OVX mice at 24h after loading compared with their non-loaded contra-lateral controls.

Table CL4 Fold change of differentially expressed genes in WT-SN mice at 24h after loading compared with their non-loaded contra-lateral controls.

Table CL5 Fold change of differentially expressed genes in ERα^-/-^ mice at 3h after loading compared with their non-loaded contra-lateral controls.
